# Mapping the Risk of Soil-Transmitted Helminthic Infections in the Philippines

**DOI:** 10.1371/journal.pntd.0003915

**Published:** 2015-09-14

**Authors:** Ricardo J. Soares Magalhães, Maria S. Salamat, Lydia Leonardo, Darren J. Gray, Hélène Carabin, Kate Halton, Donald P. McManus, Gail M. Williams, Pilarita Rivera, Ofelia Saniel, Leda Hernandez, Laith Yakob, Stephen T. McGarvey, Archie C. A. Clements

**Affiliations:** 1 School of Veterinary Science, The University of Queensland, Gatton Campus, Gatton, Australia; 2 Children’s Health and Environment Program, Queensland Children’s Medical Research Institute, The University of Queensland, Herston Campus, Herston, Australia; 3 University of Queensland, Infectious Disease Epidemiology Unit, School of Population Health, Herston, Queensland, Australia; 4 University of the Philippines Manila, College of Public Health, Department of Parasitology, Manila, Philippines; 5 Research School of Population Health, The Australian National University, Canberra, Australian Capital Territory, Australia; 6 Department of Biostatistics and Epidemiology, College of Public Health, University of Oklahoma Health Sciences Center, Oklahoma City, Oklahoma, United States of America; 7 Institute for Health and Biomedical Innovation, Queensland University of Technology, Kelvin Grove, Queensland, Australia; 8 Infectious Disease Division, QIMR-Berghoffer Medical Research Institute, Herston, Queensland, Australia; 9 University of the Philippines Manila, College of Public Health, Department of Epidemiology and Biostatistics, Manila, Philippines; 10 Philippine Department of Health, National Center for Disease Prevention and Control, Manila, Philippines; 11 London School of Hygiene & Tropical Medicine, Department of Disease Control, London, United Kingdom; 12 International Health Institute, Brown University, Providence, Rhode Island, United States of America; Swiss Tropical and Public Health Institute, SWITZERLAND

## Abstract

**Background:**

In order to increase the efficient allocation of soil-transmitted helminth (STH) disease control resources in the Philippines, we aimed to describe for the first time the spatial variation in the prevalence of *A*. *lumbricoides*, *T*. *trichiura* and hookworm across the country, quantify the association between the physical environment and spatial variation of STH infection and develop predictive risk maps for each infection.

**Methodology/Principal Findings:**

Data on STH infection from 35,573 individuals across the country were geolocated at the barangay level and included in the analysis. The analysis was stratified geographically in two major regions: 1) Luzon and the Visayas and 2) Mindanao. Bayesian geostatistical models of STH prevalence were developed, including age and sex of individuals and environmental variables (rainfall, land surface temperature and distance to inland water bodies) as predictors, and diagnostic uncertainty was incorporated. The role of environmental variables was different between regions of the Philippines. This analysis revealed that while *A*. *lumbricoides* and *T*. *trichiura* infections were widespread and highly endemic, hookworm infections were more circumscribed to smaller foci in the Visayas and Mindanao.

**Conclusions/Significance:**

This analysis revealed significant spatial variation in STH infection prevalence within provinces of the Philippines. This suggests that a spatially targeted approach to STH interventions, including mass drug administration, is warranted. When financially possible, additional STH surveys should be prioritized to high-risk areas identified by our study in Luzon.

## Author Summary

Soil-transmitted helminth (STH) infections with *A*. *lumbricoides*, *T*. *trichiura* and hookworms are endemic in all 80 provinces of the Philippines, but the spatial variation in the prevalence of these infections has not been previously described. This analysis revealed that while *A*. *lumbricoides* and *T*. *trichiura* infections were widespread and highly endemic, hookworm infections were more circumscribed to smaller foci in the Visayas and Mindanao. The results also suggest that it may be necessary to place greater emphasis on improving the provision of water, sanitation and the promotion of behavioral change for improved hygiene for the control and prevention of STH infections, particularly for hookworm.

## Introduction

Soil-transmitted helminth (STH) infections are believed to affect two billion people worldwide, equating to approximately one-third of the world’s population. *Ascaris lumbricoides*, *Trichuris trichiura* and the hookworms *Necator americanus* and *Ancylostoma duodenale*, are the species responsible for most of these infections [1]. The infective stages of these parasites are found in fecally contaminated environments, which means that a lack of clean water, sanitation and hygiene (WASH) contribute considerably to their transmission [2].

While most STH infections are asymptomatic, heavier infections can result in abdominal pain, diarrhea, malaise, and weakness. STH infections in children have also been linked to anaemia and impaired cognitive and physical development [3–5].

These STH species are widely distributed in tropical and subtropical areas [6]. In the Philippines, prevalence data from the first national baseline survey of STH infections conducted from 2005 to 2008 showed endemic levels in the three regions of the country (Luzon, Visayas, and Mindanao) [7, 8] To address the problem of STH infections, the Philippines Department of Health has been implementing the Integrated Helminth Control Program since 2006 [9]. The program has chemotherapy with albendazole or mebendazole as its cornerstone and targets all children aged 12 months to 12 years, and other special population groups including pregnant women, adolescent females, farmers, and indigenous populations across the Philippines [9, 10]. In addition, people found to be infected with any of the STH species are treated. Anthelminthic drugs are provided free of charge. The control program also includes installation of water and sanitation facilities and educational approaches to improve hygienic practices [11].

To guide STH control programs and ensure that scarce resources are allocated efficiently, it is important to know which areas have the highest prevalence of infection. Traditional ways of implementing and evaluating parasite control programs often entail significant costs that restrict their use in developing countries like the Philippines. Previous mapping efforts of STH prevalence in Southeast Asia enabled the construction of models to predict areas of high prevalence, the estimation of the number of people needing treatment and the identification of intervention targets for national helminth control programs [12]. In recent years, spatial prediction methods that employ model-based geostatistics (MBG) have been developed to guide parasite control programs [13]. These methods can be used to generate species-specific predictive prevalence maps, and to estimate relationships and associated uncertainty between infection outcomes and covariates, including environmental factors such as rainfall and temperature. Imperitive to these highly aggregated parasitic diseases, MBG also has the capacity to account for the inherent clustering of infection. Examples of the application of the MBG approach to understand STH epidemiology are available for sub-Saharan Africa [13–15] and more recently in South America [16] but none have been developed in the Southeast Asian context.

This study aimed to quantify the association between the physical environment and the prevalence of *A*. *lumbricoides*, *T*. *trichiura* and hookworm infections in the Philippines and to generate statistically robust spatial predictions of STH infections for the Philippines.

## Materials and Methods

### Ethics statement

Ethical clearance for this analytical study was provided by the University of Queensland Human Research Ethics Committee (Project Number 2011000692). This analytical study utilised STH data collected in the National Parasitological Survey and data collected in a study in Western Samar; the research ethics procedures for both these studies are detailed elsewhere [7, 8, 17]. All data used in the study was anonymized.

### Data for analysis

We used STH data collected during the most recent (conducted from 2005–2007) national schistosomiasis survey in the Philippines; study design and data collection procedures were described in detail elsewhere [7, 8]. In brief, the national survey used stratified two-stage systematic cluster sampling whereby stratification was done by region and then by prevalence level. The endemic provinces were initially divided into high prevalence, moderate prevalence and low prevalence groups based on the surveys conducted during the implementation of the Philippines Health Development Program in the 1990’s. The province was the primary sampling unit and the barangays the secondary sampling unit. While all known endemic provinces were included in the sampling population, among the non-endemic provinces, random selection was performed. The barangays were selected proportional to population size. Households within barangays were selected in a systematic manner from a master list of all the households in the barangay. Two stool samples were collected on separate days from each study participant. However, the submission of the second stool was erratic and only the result from the first examination was taken. The Kato-Katz thick smear was used to detect eggs of *A*. *lumbricoides*, *T*. *trichiura*, and hookworms. Demographic data including age and sex were also collected for each participant. The data from the Visayas was supplemented with data from the cross sectional component of a cohort study that included 5,624 residents of 50 barangays from Western Samar which had not been included in the national survey [17]. The purpose of the Samar study was to determine the effect of water management systems and non-human animal hosts on *S*. *japonicum* transmission dynamics and their role in human infection parameters [18, 19]. The villages were sampled to represent 25 mainly rain-fed and 25 mainly irrigated villages. In each selected village, 35 households with at least one rice farmer were selected at random, and at most 6 persons per household, including at least one rice farmer, were selected at random. This dataset included test results for three consecutive days but to be consistent with available data from the national survey only data from the first day was used in the analysis.

### Georeferencing of barangays

**Fig 1:**
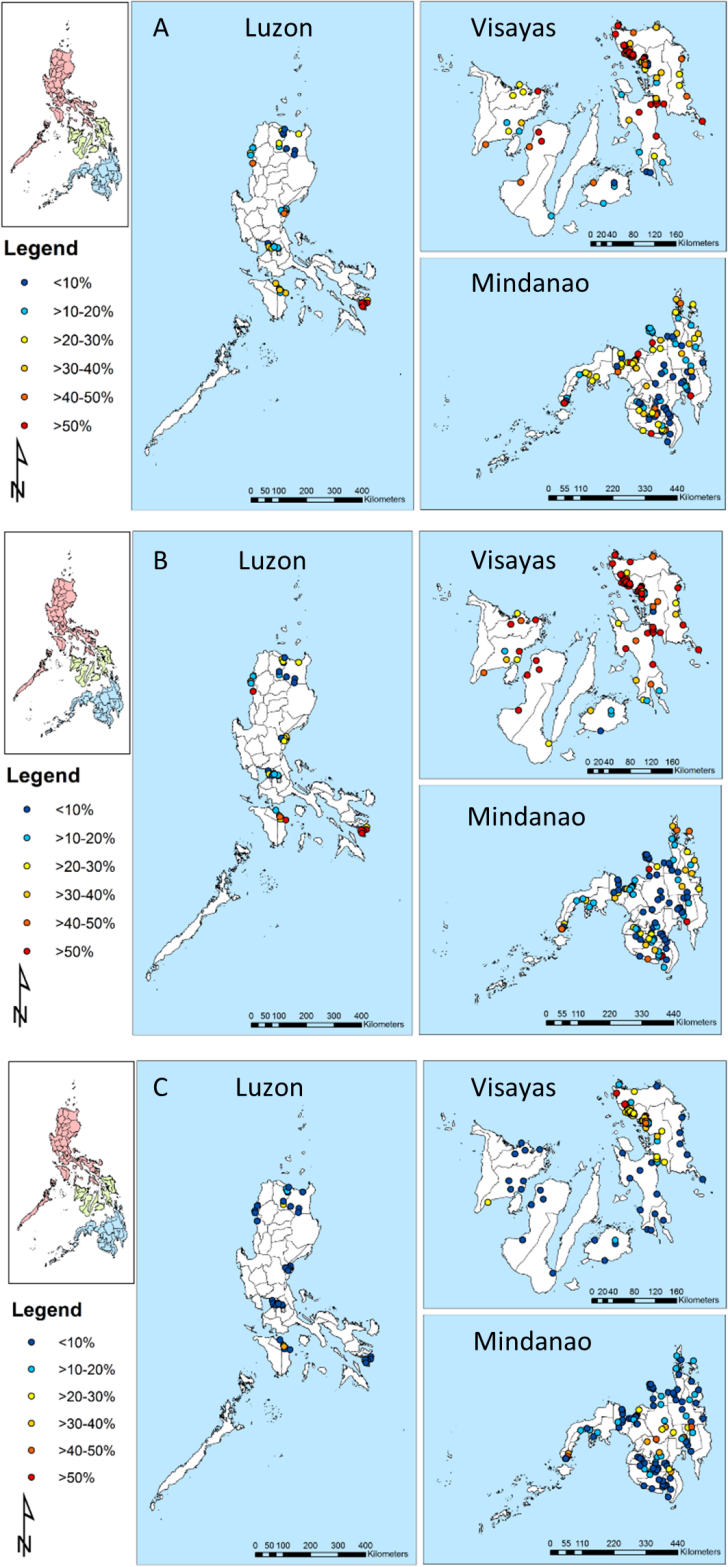
Geographical distribution of survey locations and observed prevalence for *Ascaris lumbricoides* (A), *Trichuris trichiura* (B) and hookworm (C). Note: Luzon–left, Visayas–top right and Mindanao–bottom right.

The unit of analysis was the barangay, the smallest administrative unit in the Philippines. The mean length of the longest axis of barangays was 11km (SD:10.3). Barangay centroids were estimated using the geographical information system (GIS) software QuantumGIS (QGIS) version 1.7.3 (QGIS Development Team, 2011). This procedure was based on combined information from shapefiles of the barangays of the Philippines, which were obtained from the geographic data warehouses DIVA GIS (www.diva-gis.org/Data) and PhilGIS (www.philgis.org) for the Philippines. A total of 214 barangays in Luzon, the Visayas and Mindanao were included in the analysis (Fig 1).

### Data on the physical environment

Remotely sensed environmental data for land surface temperature, rainfall and NDVI and distance to perennial water bodies (DPWB) were obtained from WorldClim (www.worldclim.org). Land surface temperature (LST) was considered in the analysis because *A*. *lumbricoides*, *T*. *trichiura* and hookworm have thermal thresholds outside of which the survival of the infective stages in the soil declines [1]. Rainfall and DPWB were also considered because these will affect the moisture of the soil where the helminth infective stages are found, and therefore their survival. Normalized difference vegetation index (NDVI) which serves as a proxy measure of rainfall for a 1 km × 1 km grid cell resolution were obtained from the National Oceanographic and Atmospheric Administration’s (NOAA) Advanced Very High Radiometer and was also included in the analysis. Using QGIS, the values of these environmental co-variates were extracted for each barangay.

### Variable selection and residual spatial variation

For the purpose of the analysis the presence of parasite eggs in stool identified by the Kato-Katz method was used as the outcome variable and thus all individuals were categorized into infected and not infected based on the presence of at least one egg. Initial variable selection included age and sex because these two factors have been shown to be associated with STH infections probably by influencing exposure and susceptibility to infection [1]. The environmental variables (rainfall, DPWB, LST and NDVI) were also considered in the initial variable screening stage. Correlations between environmental covariates were investigated using Pearson’s correlation coefficients. Scatter plots were used to assess the relationship between the barangay-level STH prevalence and the value of each of the environmental variables. Multivariable logistic regression models for a Bernoulli-distributed outcome, with cluster correction by barangay using robust standard errors, were built for each STH species for each region of the Philippines, i.e., Luzon, the Visayas, and Mindanao using the statistical software Stata version 10.1 (Stata corporation, College Station, TX). Residuals of the final non-spatial models were examined for spatial autocorrelation by generating a semivariogram using the geoR package of R software v.2.15. One semivariogram was generated for each STH species for each region of the Philippines to determine how much of the clustering of STH infections is explained by location, and to establish the propensity and size of geographical clusters.

### Spatial risk prediction and model validation

**Fig 2:**
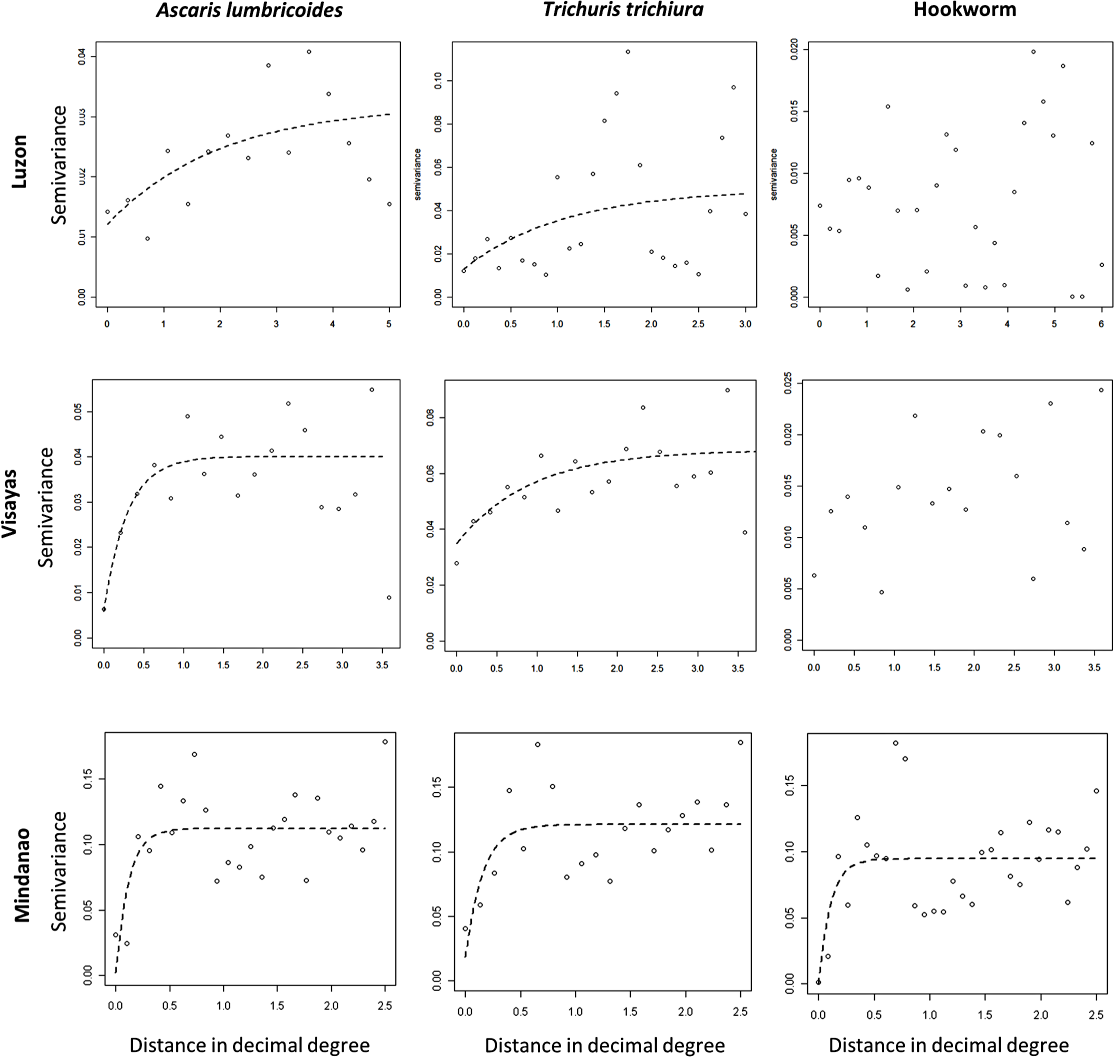
Residual semivariograms for *Ascaris lumbricoides*, *Trichuris trichiura* and hookworm in Luzon, the Visayas and Mindanao. Note: distance is recorded in decimal degrees (1 decimal degree is 111km at the equator).

Bayesian logistic geostatistical models were built for each STH species for the regions of Luzon and the Visayas combined and for the region of Mindanao separately using WinBUGS (MRC Biostatistics Unit, Cambridge, and Imperial College London, UK). This decision was based on the results of the semivariogram analysis (Fig 2), which indicated similar spatial dependence in STH prevalence in the regions of Luzon and the Visayas. The models included an intercept, the individual level variables age (categorized into children aged <5 years and 5–19 years, and adults aged >20 years) and sex, the environmental variables DPWB, LST, NDVI and rainfall, and a geostatistical random effect (S1 Text). In addition, the model included adjustment for diagnostic uncertainty by modeling sensitivity and specificity as random variables. The Kato-Katz technique is widely used for detecting helminth eggs in stools. The sensitivity of the test is influenced by changes in the number of eggs excreted in the feces from day to day. True prevalence was modeled as a function of the observed prevalence and test sensitivity and specificity, with the generalised linear model fit to the true prevalence parameter. Priors for the sensitivity and specificity were specified as beta distributions; we used the alpha and beta parameters reported in previous studies [20] (Table A in S1 Text). The covariate effects were summarized using the mean and 95% credible intervals (representing the range of values that contains the true value with a probability of 95%); a significant result for a coefficient is indicated by where the 95% credible interval does not cross zero. The geostatistical random effect modeled spatial correlation as a function of the separating distance between pairs of barangays. Model predictions for *A*. *lumbricoides* and *T*. *trichiura* were used to generate representative STH risk maps for males aged 5–19 years (the subgroup with the highest prevalence for these STH) and model predictions for hookworm were used to generate representative STH risk maps for males aged >20 years (the subgroup with the highest risk for this STH) across the Philippines in ArcGIS version 10.0. Note the overall mean predicted prevalence is specific to the age and sex group (i.e. choice of a different age-sex group would result in a different spatial mean), with spatial variation around the mean being influenced by the environmental variables and the spatial correlation component of the model. This means that the relative differences between locations are consistent and so the maps presented are representative of the spatial distribution of risk for all age groups and both sexes. The priors used for the model parameters (spatial and non-spatial) are given in the S1 Text. To determine the discriminatory performance of the model predictions relative to observed prevalence thresholds (20% and 50%; corresponding to prevalence thresholds for WHO-recommended STH control strategies [21]), the area under the curve (AUC) of the receiver operating characteristic was used (more detail in Table B in S1 Text). An AUC value of >0.7 was taken to indicate acceptable predictive performance [22]. We estimated the mean prediction error and the percentage of the overall observed mean attributable to the error estimate.

## Results

### Data for analysis

For the purpose of the spatial modeling, STH data collected during the most recent (conducted from 2005–2007) national schistosomiasis survey in the Philippines were supplemented with data from from Western Samar (in the Visayas) which had not been included in the national survey. In total, we included data from 35,573 participants from 214 barangays in Luzon, Visayas, and Mindanao (Fig 1). This corresponded to 2,701 individuals in Luzon, 13,203 individuals in the Visayas and 19,669 individuals in Mindanao with complete information regarding STH infection status, barangay geolocation and demography (i.e. age and sex) (Table 1). The mean observed prevalence of *A*. *lumbricoides* was 23.7% for Luzon, 38.4% for the Visayas and 21.2% for Mindanao. For *T*. *trichiura*, the mean observed prevalence was 27.9% for Luzon, 53.6% for the Visayas and 16.8% for Mindanao. The mean observed prevalence of hookworm was 4.5% for Luzon, 18% for the Visayas and 11.3% for Mindanao.

**Table 1:** Characteristics of 35,573 individuals and properties of the physical environment of survey locations included in the analysis.

Variable	Luzon	Visayas	Mindanao
**Number of survey locations**	36	93	108
**Number of individuals tested**	2,701	13,203	19,669
***A*. *lumbricoides* infection**			
*Yes*	640 (24%)	5,065 (38%)	4,154 (21%)
*No*	2,061	8,138	15,515
***T*. *trichiura* infection**			
*Yes*	754 (28%)	7,074 (54%)	3,311 (17%)
*No*	1,947	6,129	16,358
**Hookworm infection**			
*Yes*	122 (4.5%)	2,386 (18%)	2,225 (11%)
*No*	2,579	10,817	17,444
**Age in years**			
*Mean*	23.6	25.1	24.4
*SD*	19.5	19.9	18.6
**Sex**			
*Male*	1,272 (47%)	6,556 (50%)	9,605 (49%)
*Female*	1,429	6,647	10,064
**Rainfall (mm)**			
*Mean*	314.22	273.19	213.78
*SD*	127.53	6.00	55.43
**Land surface temperature (C°×10)**			
*Mean*	275.08	245.79	260.67
*SD*	10.27	61.64	14.63
**Normalised Difference Vegetation Index**			
*Mean*	1,493.45	1,546.77	1,545.80
*SD*	148.97	93.86	72.16
**Distance to perennial water bodies (km)**			
*Mean*	16.7	30.0	38.9
*SD*	15.5	22.2	33.3

### Residual spatial dependence

*A*. *lumbricoides* and *T*. *trichiura* prevalence proportions showed a tendency for clustering in all three regions, unlike hookworm prevalence proportions which showed spatial clustering only in Mindanao (Fig 2). The greatest tendency for clustering was exhibited by *T*. *trichiura* in Mindanao (Fig 2H) and the largest estimated cluster size was for *T*. *trichiura* in Luzon (Fig 2B).

### Spatial risk prediction

Model results (Table 2 for Luzon and the Visayas and Table 3 for Mindanao) indicated that individuals aged 5–19 years had higher prevalence of infection than individuals aged <5 years for all three STH. In contrast, the prevalence of infection was higher among individuals aged >20 years as compared to those aged <5 years for *T*. *trichuri*a and hookworm, but not for *A*. *lumbricoides*. In Luzon and the Visayas, males had higher prevalence of *A*. *lumbricoides* infection compared with females. While in Luzon and the Visayas females had higher hookworm infection prevalence compared to males, in Mindanao males had higher hookworm infection prevalence compared to females. Environmental factors influenced the prevalence of the three parasites differently in the three regions. In Luzon and the Visayas, only the distance to water bodies was associated with an increase in the prevalence of hookworm infections. No other environmental variable was associated with the prevalence of the three parasites. In contrast, in Mindanao, land surface temperature was associated with an increase of the prevalence odds of *A*. *lumbricoides* and *T*. *trichuria*, and was associated with a decrease in prevalence of hookworm infections. Similarly, NDVI was associated with an increase in the prevalence of hookworm infections and was associated with a decrease of *A*. *lumbricoides* and *T*. *trichuria*. Rainfall was associated with an increased prevalence of *T*. *trichuria* and distance to water bodies was associated with an increase of the prevalence of hookworm, the latter being similar to that observed in Luzon and the Visayas.

**Table 2:** Estimates of model parameters (in the log odds scale) for STH prevalence in Luzon and Visayas, Philippines, based on Bayesian geostatistical logistic regression models.

	*Ascaris lumbricoides*	*Trichuris trichiura*	Hookworm
Variable	Posterior mean	Posterior mean	Posterior mean
	(95%Credible Interval)	(95%Credible Interval)	(95%Credible Interval)
*5–19 years (vs <5 years)*	0.20 (0.02, 0.37)	0.84 (0.39,1.34)	1.94 (1.15, 2.82)
*>20 years (vs <5 years)*	-0.26 (-0.46, -0.07)	0.52 (0.07,1.02)	3.69 (1.94, 5.86)
*Female (vs Male)*	-0.44 (-0.60, -0.28)	0.09 (-0.29,0.46)	5.54 (3.39, 7.97)
Distance to water bodies*	0.08 (-0.13, 0.32)	-0.20 (-0.72,0.48)	5.25 (0.92, 11.01)
Land surface temperature*	-0.21 (-0.54, 0.08)	0.21 (-0.58,0.98)	0.43 (-2.33, 3.54)
NDVI*	0.15 (-0.19, 0.55)	0.52 (-0.47,1.66)	-1.18 (-4.25, 3.62)
Rainfall*	-0.03 (-0.40, 0.31)	0.11 (-1.40,1.43)	0.77 (-5.00, 5.24)
*Intercept*	-0.78 (-1.34, -0.06)	-4.76 (-7.54,-1.74)	-15.14 (-22.83, -10.65)
**Spatial effects**			
*Rate of decay of spatial autocorrelation [Phi (φ); in decimal degrees* ^*-1*^ *]*	11.91 (5.12, 19.09)	1.55 (0.42,4.44)	5.80 (1.30, 14.6)
*Variance of spatial random effect*	1.83 (1.20, 2.94)	21.53 (6.93,56.07)	29.15 (5.82, 97.66)

**Table 3:** Results of model parameters (in the log odds scale) for STH prevalence in Mindanao, Philippines, based on Bayesian geostatistical logistic regression models.

	*Ascaris lumbricoides*	*Trichuris trichiura*	Hookworm
Variable	Posterior mean	Posterior mean	Posterior mean
	(95%Credible Interval)	(95%Credible Interval)	(95%Credible Interval)
*5–19 years (vs <5 years)*	0.24 (0.06, 0.41)	0.63 (0.29,1.11)	1.04 (0.35, 2.82)
*>20 years (vs <5 years)*	-0.05 (-0.09,-0.02)	0.42 (0.07,0.96)	2.06 (1.13, 3.87)
*Female (vs Male)*	0.11 (-0.66,1.01)	-0.06 (-0.88,0.71)	-1.12 (-1.93, -0.02)
Distance to water bodies*	0.33 (-0.49,1.19)	0.79 (-0.71,2.22)	2.94 (1.53, 4.12)
Land surface temperature*	0.79 (0.06,1.65)	1.65 (0.31, 3.59)	-0.75 (-1.78, -0.01)
NDVI*	-1.02 (-2.48,-0.02)	-1.40 (-2.61, -0.32)	2.27 (0.17, 3.85)
Rainfall*	0.92 (-0.22,1.70)	1.22 (0.06, 2.86)	-0.48 (-1.46, 0.79)
*Intercept*	-0.84 (-4.61,3.59)	17.04 (7.88, 24.32)	-1.03 (-4.72, 1.34)
**Spatial effects**			
*Rate of decay of spatial autocorrelation [Phi (φ); in decimal degrees* ^*-1*^ *]*	11.81 (2.53,19.71)	3.90 (0.60, 9.60)	1.48 (0.22, 4.60)
*Variance of spatial random effect*	16.86 (6.83,36.21)	29.78 (6.90,111.87)	59.44 (17.48,226.5)

The parameter Phi (*φ*) refers to the rate of decay of spatial autocorrelation and indicates the size of clusters. The radius of a cluster in kilometers corresponds to (3/φ)*111 (note: one decimal degree is equivalent to approximately 111 km at the equator). For *A*. *lumbricoides* infection, the radii of the clusters measured 28 km in all regions. For *T*. *trichiura* infection, the clusters’ radii measured 215 km in Luzon and Visayas and 85 km in Mindanao. For hookworm infection, the radius of the clusters was 57 km in Luzon and Visayas and 225 km in Mindanao. The tendency for spatial clustering was the strongest for hookworm in all regions (the higher value the spatial variance parameter the higher the tendency for spatial clustering) (Table 2 and Table 3).

**Fig 3:**
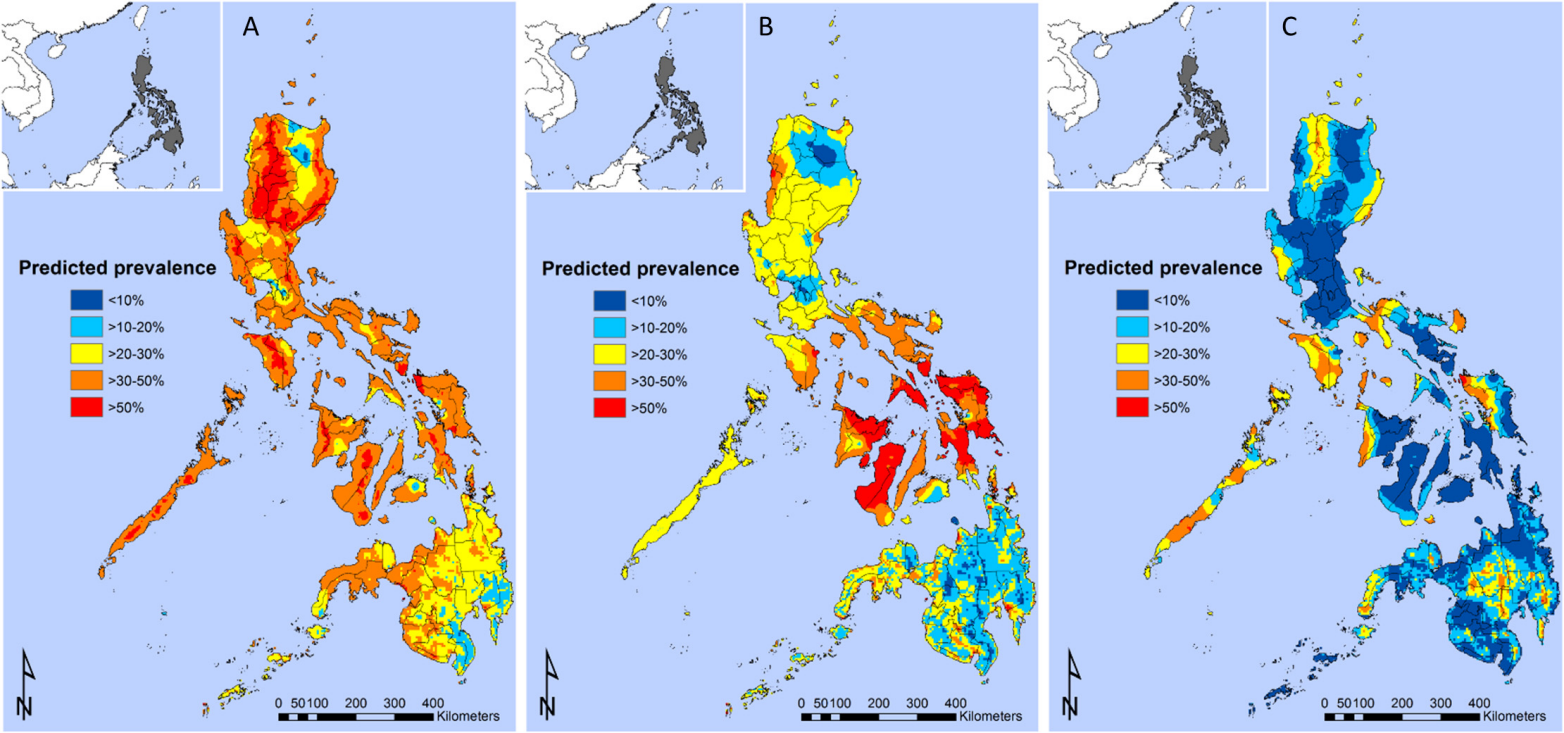
Predicted spatial distribution of *Ascaris lumbricoides* (A), *Trichuris trichiura* (B) and hookworm (C) for boys aged ≥20 years for the Philippines.

The predicted geographical distribution of *A*. *lumbricoides* infection indicated that it was widespread and endemic in Luzon and Visayas, with areas of high prevalence (>50%) predicted for many locations in these two regions (Fig 3A). *T*. *trichiura* was particularly widespread and highly endemic in the Visayas (Fig 3B). Hookworm was much more focal compared with *A*. *lumbricoides* and *T*. *trichiura* in that areas of predicted high prevalence (>50%) of hookworm were circumscribed to Zambales in the central region, Isabela in the Cagayan Valley, Apayao in the Cordillera region, north of the Bicol region, central areas of the Mindoro island, parts of Palawan island, western Samar in the island of Samar and in Antique in the island of Panay (Fig 3C). In Luzon and the Visayas, many areas of high prevalence of *A*. *lumbricoides* and *T*. *trichiura* overlapped. In Mindanao, *A*. *lumbricoides* overlapped with *T*. *trichiura* in the Zamboanga Peninsula to the west of the island. In Mindanao, most areas were predicted to reach prevalences between 10–40% for *A*. *lumbricoides*, while *T*. *trichiura* was more circumscribed with areas predicted to be highly endemic in small foci in Surigao del Norte and the Compostela Valley in the Davao region. Hookworm was predicted to have a prevalence of between 20–40% in the central provinces of Mindanao, including Cotabato, Bukidnon, Agusan del Sur and Davao.

## Discussion

This study represents the first to use model-based geostatistical predictive methods for STHs in a Southeast Asian context and demonstrates that STH infection prevalence in the Philippines is spatially variable. This study also shows that the predicted geographical distribution of *A*. *lumbricoides* and *T*. *trichiura* infection prevalence is more widespread than hookworm infection and in many areas the two species overlap. Finally, it indicates that the measured environmental factors are more strongly associated with the prevalence of these parasites in Mindanao than in Luzon and the Visayas.

The finding that *A*. *lumbricoides* and *T*. *trichiura* infections are more prevalent in individuals aged 5–19 years compared with other age groups is consistent with the known epidemiology of these infections. Previous studies show that the relationship between age and prevalence of *Ascaris* and *Trichuris* infections are characteristically convex in shape with the highest intensities observed in school-aged children [5]. This is likely related to behaviour (playing in the soil and possibly unsatisfactory hygiene habits) that leads to exposure to the parasites’ infective stages. Our study also shows that in the case of hookworm, individuals aged ≥20 years had significantly higher prevalence of infection compared with other age groups, likely reflecting cumulative occupational exposures in older individuals such as farming. These are consistent with previous studies in Southeast Asia and elsewhere that describe how hookworm prevalence rises with increasing age to a plateau in adulthood probably due to the cumulative exposure to fecal-contaminated environments brought about by agricultural activities [5, 12]. The results of our study show that the effect of sex on STH infection is less obvious and differs between regions of the Philippines. In agreement with previous studies, we found that males are at significantly higher risk of infection compared with females for *A*. *lumbricoides* in Luzon and the Visayas and for hookworm in Mindanao [12, 23, 24]. The effect size of males is strongest for hookworm in Mindanao, presumably because a substantial portion of the land area there is utilized for agriculture and in this region males are traditionally more involved in agricultural occupations than females. Interestingly, the relationship between sex and hookworm infection are opposite in Luzon and Visayas, with females being positively associated with hookworm infection, a feature warranting further investigation.

This study also shows that the effect sizes of environmental properties, such as distance to perennial water bodies, land surface temperature, NDVI (an index of vegetation) and rainfall in STH infection are generally greater in Mindanao compared with Luzon and the Visayas. In Luzon and the Visayas only distance to water bodies was associated with hookworm infection suggesting that, unmeasured, small scale factors such as socioeconomic status and behavior may be important predictors of the spatial variation in STH infection in this region. In both regions, the findings support the role of the distance to water bodies in hookworm infection suggesting that the presence of infection increases further from the water bodies. In addition, the effect size for the proximity to water bodies was larger in Luzon and the Visayas as compared to that seen in Mindanao suggesting that, in addition to occupational exposure, the lower socio-economic status and reluctance to seek treatment in communities of Luzon and the Visayas may also be an important predictor of hookworm infection in this region. While increasing land surface temperature in Mindanao was shown to be associated with the increasing prevalence of *A*. *lumbricoides* and *T*. *trichiura*, the relationship was reversed for hookworm. This difference may be partly explained by the known characteristics of the hookworm lifecycle involving the survival and development of mobile larval stages in the soil, which seek optimal conditions of temperature and humidity. In addition, the results for Mindanao demonstrate that increasing vegetation indexes were associated with increased prevalence of hookworm, suggesting a potential role of land use related to agricultural activities on the transmission of hookworm. Notably, this effect was reversed for *A*. *lumbricoides* and *T*. *trichiura*.

The significant unexplained variation in STH prevalence related to survey location (as assessed by the residual semivariogram), particularly that estimated for Mindanao, suggested that considerable STH clustering was left unaccounted for by variables included in the non-spatial multivariable model. This finding justified the need for formally modelling spatial clustering across different regions in the Philippines using model-based geostatistics. The fact that The Philippines is an archipelago presents a technical challenge to the application of MBG methods, but the decision to model risk as a spatially continuous process across the three island groups was justified on the following grounds. First, it is impractical to model risk, or the spatial correlation structure, separately by island because of the thousands of islands in the Philippines archipelago–pragmatically, we chose to model risk separately by the three main island groups. Secondly, inter-island travel is extremely frequent in the Philippines and we consider islands in close proximity to be intimately connected. A major advantage of our approach is that it adjusted for the low sensitivity of the Kato–Katz thick smear examination in the modelling framework and produces a more accurate assessment of prevalence. The low sensitivity of Kato-Katz has been particularly prominent in low infection intensities and due to day-to-day variation in egg output of the adult worms [25]. While the Kato–Katz remains the cheapest and often the only available method in the field, its lack of sensitivity means that survey results are likely to underestimate the ‘true’ prevalence, and adjustments for measurement error should be taken into account [20].

The STH predictive prevalence maps demonstrate that almost the entire area of the Philippines is endemic for at least one STH, warranting nationwide control. The predicted prevalence of *A*. *lumbricoides* and *T*. *trichiura* show that these infections are highly endemic and have a widespread distribution. The results are consistent with the known ubiquity of *A*. *lumbricoides* and *T*. *trichiura* infections in the Philippines and the similar mode of transmission of the two species, i.e., the ingestion of embryonated eggs. The findings also show the value of updating the current database of STH for the Philippines with new data; additional data for Western Samar has allowed the identification of areas which could not have been detected had the analysis been carried out on national survey data alone. While areas with the highest predicted prevalence of *A*. *lumbricoides* are located in Luzon, areas with the highest predicted prevalence of *T*. *trichiura* are located in the Visayas, in line with previous smaller scale studies [26–28]. Our results also show extensive areas of high endemicity in the Philippines where *A*. *lumbricoides* and *T*. *trichiura* infections overlap. The maps predicting the prevalence of hookworm differed markedly from those of the other STH, showing a focal distribution of the parasite (corroborated by the highest tendency for clustering), with most of the areas with a high predicted prevalence in the inland areas of Mindanao, such as the Compostela Valley and more circumscribed areas in Northern Luzon and Western Samar.

Our models also predicted the presence of STH infection in areas not represented in the survey data but which historically are known to be endemic [29, 30]. This finding indicates that these can be environmentally suitable for the presence of infection. For example, our map predicts an extensive area of moderate (20–30%) to high (>50%) risk of infection for most of Palawan, indicating its environmental suitability for the presence of STH infection, which should be further investigated.

The Philippines Integrated Helminth Control Program has been in place since 2006 and has provisions for mass targeted and selective deworming. As indicated by the maps of predicted STH infection prevalence, there remain numerous areas that still have a high prevalence of STH infections. In these places, periodic treatment using albendazole or mebendazole remains essential. The coverage of the control program must also be considered. While school-based periodic administration of anti-helminthic drugs is part of the Philippines program, this is only typically carried out in the country’s public schools, despite findings that children in private schools are also affected by STH infections [28]. The results also suggest that it may be necessary to place greater emphasis on improving the provision of water, sanitation and the promotion of behavioral change for improved hygiene for the control and prevention of STH infections. This is particularly true for hookworm, as this infection seems to be associated with occupational exposures and therefore more likely to be missed by targeting schools.

The results of the study should be interpreted in light of the studies’ limitations. First, socioeconomic factors, such as access to water and sanitation and hygiene behavior (WASH) are important predictors of the small-scale spatial variation of STH infection. While WASH indicators are measured in the Philippines as part of nationwide Demographic Health Surveys (DHS), unfortunately water and sanitation indicators were not captured in the initial survey. Future research will investigate approaches to integration of available WASH data and STH infections to determine WASH associations and changes in time in relation to STH risk.

Overall the results show that STH infections in the Philippines are a widespread, major public health problem and depending on parasite species, these show remarkable spatial variation even within known endemic areas. Further surveys should be prioritized to areas in Luzon (including Palawan), which are currently underrepresented in our database.

## Supporting Information

S1 Text Statistical notation of Bayesian geostatistical models, spatial interpolation and model validation procedures. (DOC)
