# Supplementary material for: Mapping the Risk of Soil-Transmitted Helminthic Infections in the Philippines
Source: PLoS Negl Trop Dis. 2015 Sep 14;9(9):e0003915. doi: 10.1371/journal.pntd.0003915 (PMC4569387; doi:10.1371/journal.pntd.0003915)
Supplement: S1 Text — (DOC) [file pntd.0003915.s001.doc]

**SI Text. Statistical notation of Bayesian geostatistical models for prevalence of *STH* in the Philippines.**

The number of individuals of age-sex group *j* positive with a given STH parasite in given a barangay *i* and is a binomial variable *Pos_i,j_*. The models assume a conditional Binomial model where the underlying true prevalence of STH infection$n_{i,j}$ is given by:

where *Test_i,j_* is the total number of individuals tested in location *j*. Based on the law of total probability we related the observed $p_{i,j}$and true prevalence$n_{i,j}$ via the equation:.

$$p_{i,j}=n_{i,j}*s+\left(1-n_{i,j}\right)*\left(1-t\right)$$

where *s* and *t* are the sensitivity and the specificity of Kato Katz. The sensitivity and specificity were included in the model as beta distributions – the beta distribution is parameterised by the parameters α and β. The prior values used of α and β for the sensitivity and specificity are presented in Table S1 and are based on estimates from Tarafder et al 2010 . Using these parameters, the resulting sensitivity and specificity distributions for each diagnostic performance parameter are estimated by the model and presented in Table S1.

The mean observed parasite prevalence in barangay j, age-sex group i, was modelled by:

$$logit(p_{i,j})=\alpha+\sum_{z=1}^{Z} \beta_{k}\times x_{i,j,k}+u_{i}$$

where *α* is the intercept, *β* is a matrix of coefficients and *x* is a matrix of covariates, and $u_{i,j}$are coefficients representing a geostatistical random effects. These random effects have a multinomial normal distribution, of mean zero and variance-covariance matrices defined by an isotropic powered exponential spatial correlation function:

$f(d_{ab};\varphi)=\exp\left[-(\varphi d_{ab})\right]$,

where *d_ab_* are the distances between pairs of points *a* and *b*, and $\varphi$ is the rate of decline of spatial correlation per unit of distance. Non-informative priors were used for *α* (uniform prior with bounds -$\infty$ and $\infty$) and the coefficients (normal prior with mean = 0 and precision = 1 × 10^-4^). The prior distribution of $\varphi$ was also uniform with upper and lower bounds set at 0.1 and 50. (the lower bound set to ensure spatial correlation at the maximum separating distance between survey locations was <0.5, assisting identifiability ).The precision of *u_i_* was given a non-informative gamma distribution.

**Table A.** Prior values for the coefficients of the beta distributions used in the Bayesian models for the adjustment of the sensitivity and specificity of Kato-Katz stool examination technique using one stool sample for the detection of *A. lumbricoides*, *T. trichiura* and hookworm.

| **Diagnostic performance** | **Beta distribution coefficients** | | **Posterior mean and 95%CrI of diagnostic performance** | |
| --- | --- | --- | --- | --- |
|  | *α* | *β* | Luzon and Visayas | Mindanao |
| ***Ascaris lumbricoides*** |  |  |  |  |
| *Sensitivity* | 151 | 4 | 0.97 (0.94, 0.99) | 0.97 (0.94, 0.99) |
| *Specificity* | 151 | 4 | 0.97 (0.94, 0.99) | 0.95 (0.89,0.99) |
| ***Trichuris trichiura*** |  |  |  |  |
| *Sensitivity* | 45 | 4 | 0.92 (0.83,0.98) | 0.91 (0.89,0.93) |
| *Specificity* | 65 | 4 | 0.94 (0.87,0.98) | 0.94 (0.91,0.96) |
| **Hookworm** |  |  |  |  |
| *Sensitivity* | 58 | 25 | 0.70 (0.60, 0.79) | 0.54 (0.45,0.64) |
| *Specificity* | 65 | 4 | 0.94 (0.88, 0.98) | 0.95 (0.89,0.98) |

In all STH models, a burn-in of 5,000 iterations was allowed, followed by 10,000 iterations where values for the intercept, coefficients and predicted probability of infection at the prediction locations were stored. Diagnostic tests for convergence of the stored variables were undertaken, including visual examination of history and density plots; convergence was successfully achieved after 5,000 iterations.

The predictions of the prevalence of each STH prevalence in each region were made at the nodes of a 0.1 X 0.1 decimal degree grid (approximately 11 km^2^) by interpolating the geostatistical random effect and adding it to the sum of the products of the coefficients for the fixed effects and the values of the fixed effects at each prediction location. Values of predicted prevalence of STH at unsampled locations were stored for the subgroup with the highest prevalence which in the case of *A. lumbricoides* and *T. trichiura* were males aged 5-19 years and for hookworm were males aged >20 years. The interpolation of the random effect was done using the *spatial.unipred* kriging function in WinBUGS; the *spatial.unipred* command implements Bayesian kriging [1]where the values of predicted prevalence at unsampled locations are estimated (interpolated) independently of neighbouring values, as opposed to joint prediction which is conditional on the values of neighbouring unsampled locations. Joint prediction was not considered feasible in this study due to being extremely intensive computationally.

The outputs of Bayesian models, including parameter estimates and spatial prediction at unsampled locations, are distributions termed “posterior distributions”. The posterior distributions fully represent uncertainties associated with the parameter estimates. We summarised the posterior distributions in terms of the posterior mean and 95% Bayesian credible interval (CrI), within which the true value occurs with a probability of 95%. The variables were standardised by subtracting the mean and dividing by the standard deviation. The resulting regression coefficients for these variables represent the change in terms of standard deviations in prevalence that result from a change of one standard deviation in these variables.

**Model validation procedures**

To quantify the discriminatory ability of the models, in each model the entire dataset was partitioned into four random sub-sets; the model was run in three sub-sets of data and prediction was carried out to the remaining subset containing 25% of the data. The ability of the models to predict known mean prevalence of STH infection was assessed by mean prediction error which provides a measure of the bias of the predictor. The observed values were compared to the mean of the posterior distribution of each predicted value of prevalence. The result of the validation procedures for each model is presented in Table S2.

Table B: Results of model validation procedures.

| **Model** | **AUC threshold** | | **Mean prediction error*** |
| --- | --- | --- | --- |
|  | **20%** | **50%** |  |
| ***A. lumbricoides*** |  |  |  |
| *Luzon & Visayas* | 0.61 (0.57, 0.64) | 0.66 (0.62, 0.71) | 0.05 (16.13) |
| *Mindanao* | 0.59 (0.55, 0.67) | 0.68 (0.64, 0.74) | 0.01 (4.76) |
| *T****. trichiura*** |  |  |  |
| *Luzon & Visayas* | 0.63 (0.57, 0.69) | 0.59 (0.56, 0.63) | 0.05 (12.20) |
| *Mindanao* | 0.76 (0.71, 0.80) | 0.71 (0.65, 0.77) | 0.03 (17.65) |
| **Hookworm** |  |  |  |
| *Luzon & Visayas* | 0.54 (0.49, 0.59) | 0.72 (0.65, 0.78) | 0.04 (36.36) |
| *Mindanao* | 0.83 (0.77, 0.87) | 0.82 (0.75, 0.87) | 0.02 (18.19) |

* Values in parenthesis represent the percentage of the overall mean attributed to the error estimate.

**Figure S1. Predicted standard deviation of Ascaris lumbricoides prevalence in Luzon and Visayas (A) and Mindanao (B).**

**
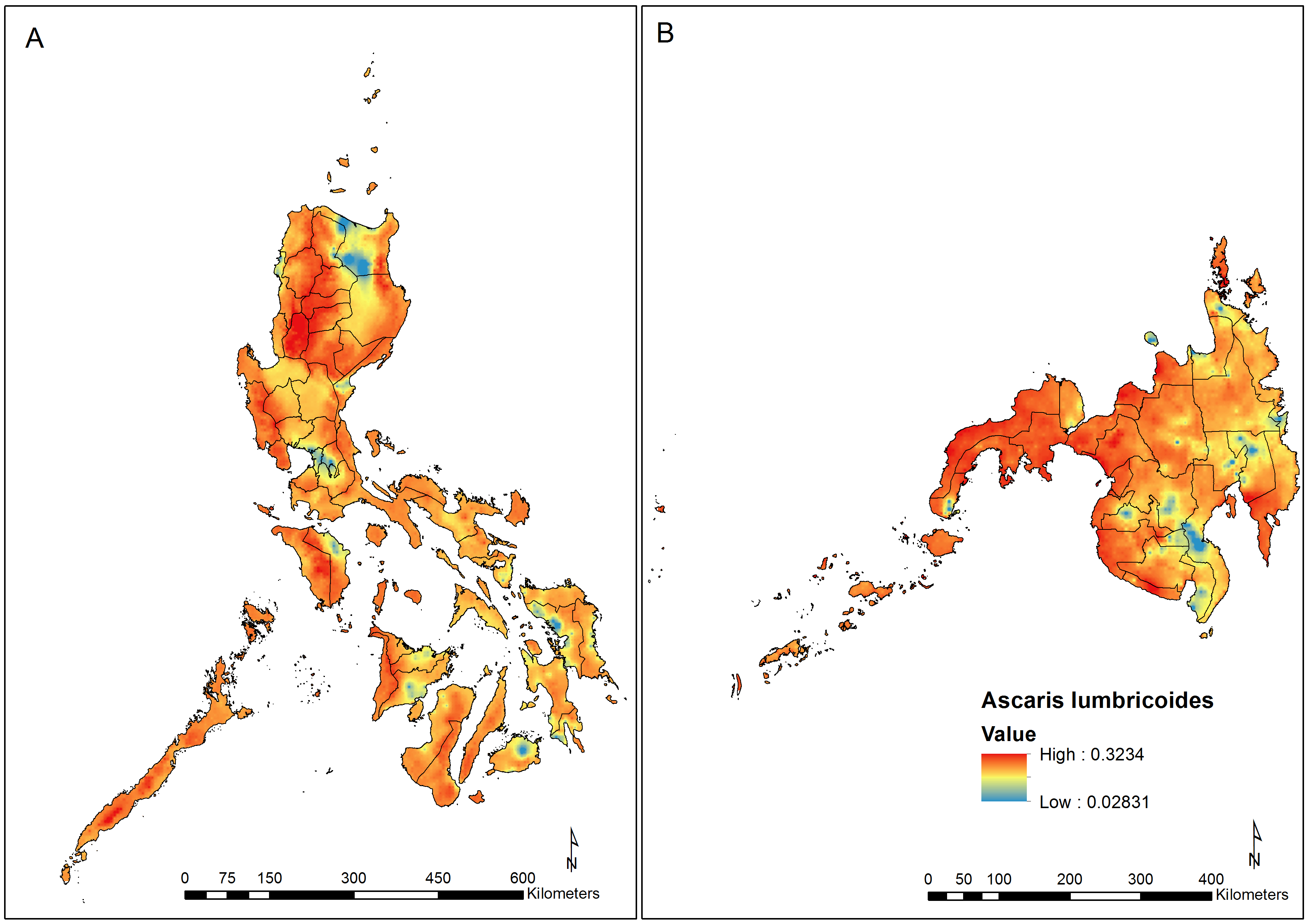
**

**Figure S2. Predicted standard deviation of *Trichuris trichiura* prevalence in Luzon and Visayas (A) and Mindanao (B).**

**
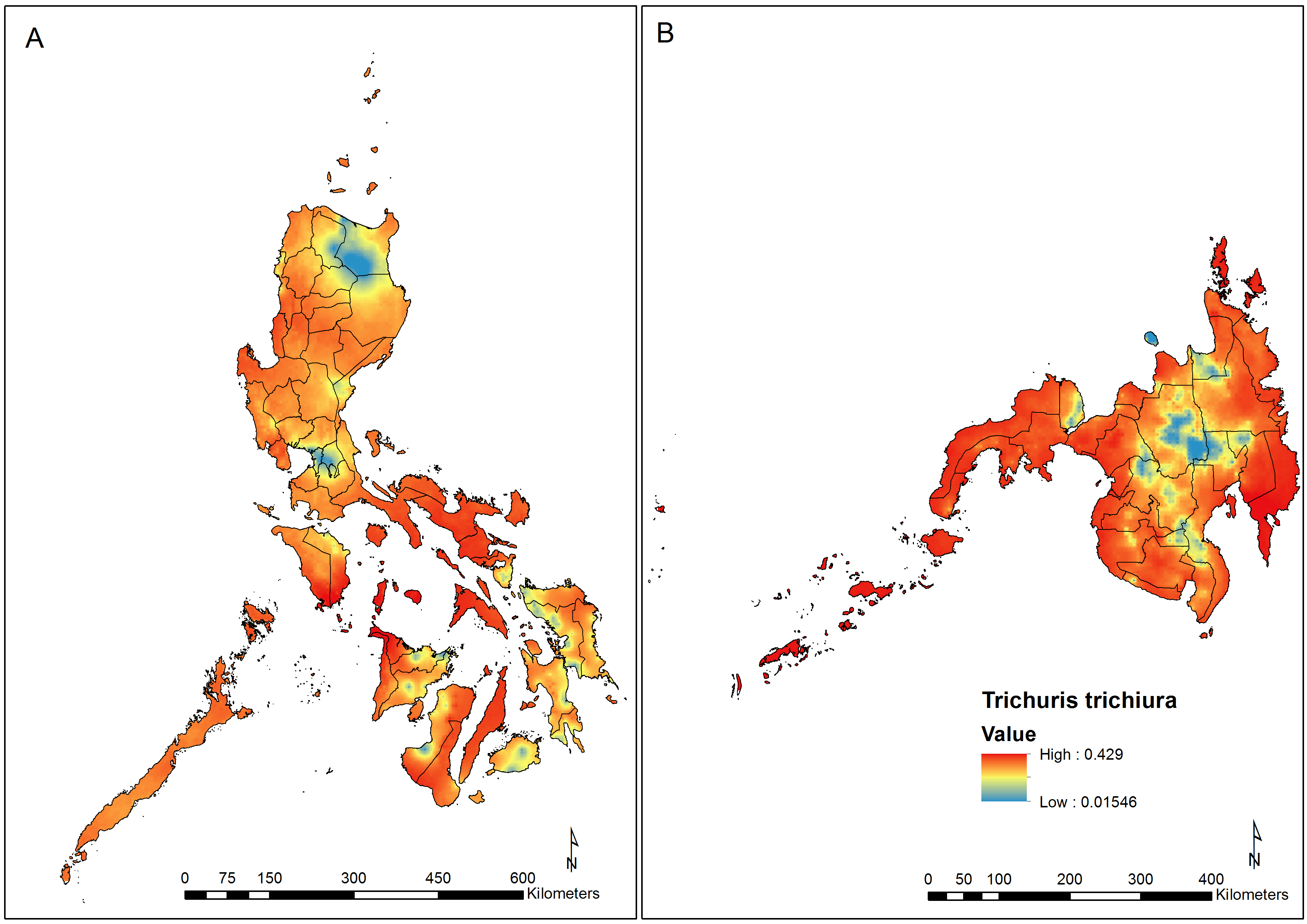
**

**Figure S3. Predicted standard deviation of hookworm prevalence in Luzon and Visayas (A) and Mindanao (B).**

**
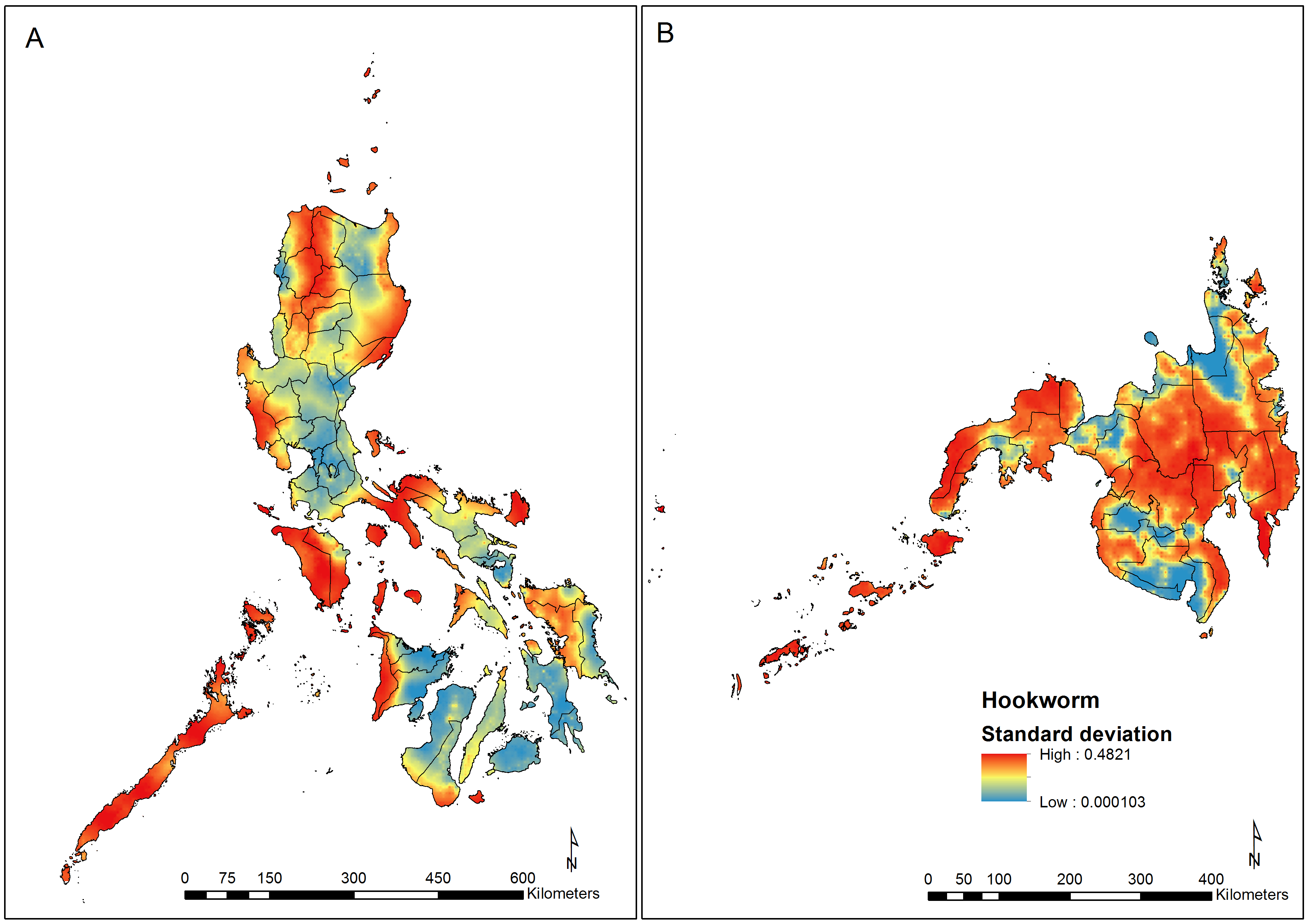
**

**References**

1. Tarafder MR*, et al.* (2010) Estimating the sensitivity and specificity of Kato-Katz stool examination technique for detection of hookworms, Ascaris lumbricoides and Trichuris trichiura infections in humans in the absence of a 'gold standard'. *Int J Parasitol* 40(4):399-404.

2. Thomas; A, Best; N, Lunn; D, Arnold; R, & Spiegelhalter D (2004) GeoBUGS User Manual. (Medical Research Council Biostatistics Unit, Cambridge).
